# Backscattering design for a focusing grating coupler with fully etched slots for transverse magnetic modes

**DOI:** 10.1038/s41598-018-36082-z

**Published:** 2018-12-10

**Authors:** Jahn Hoffmann, K. Marvin Schulz, Giampaolo Pitruzzello, Lena Simone Fohrmann, Alexander Yu. Petrov, Manfred Eich

**Affiliations:** 1 Institute of Optical and Electronic Materials, Hamburg University of Technology, Eissendorfer Str. 38, Hamburg, 21073 Germany; 2 Department of Physics, University of York, York, YO10 5DD UK; 3 ITMO University, 49 Kronverkskii Ave., 197101 St Petersburg, Russia; 4 Institute of Materials Research, Helmholtz-Zentrum Geesthacht, Max-Planck-Strasse 1, Geesthacht, D-21502 Germany

## Abstract

Grating couplers are a fundamental building block of integrated optics as they allow light to be coupled from free-space to on-chip components and vice versa. A challenging task in designing any grating coupler is represented by the need for reducing back reflections at the waveguide-grating interface, which introduce additional losses and undesirable interference fringes. Here, we present a design approach for focusing TM grating couplers that minimizes these unwanted reflections by introducing a modified slot that fulfills an anti-reflection condition. We show that this antireflection condition can be met only for the Bloch mode of the grating that concentrates in the dielectric. As a consequence the light is scattered from the grating coupler with a negative angle, referred to as “backscattering design”. Our analytic model shows that the anti-reflection condition is transferrable to grating couplers on different waveguide platforms and that it applies for both TE and TM polarizations. Our experimentally realized focusing grating coupler for TM-modes on the silicon photonics platform has a coupling loss of (3.95 ± 0.15) dB at a wavelength of 1.55 µm. It has feature sizes above 200 nm and fully etched slots. The reflectivity between the grating coupler and the connected waveguide is suppressed to below 0.16%.

## Introduction

Silicon wire waveguides can strongly confine light to the nanoscale and exhibit a low propagation loss enabling long-range, on-chip guiding of light. The high level of integration of photonic waveguides on the CMOS compatible silicon platform is recognized as a disruptive technology that enables next-generation communications systems, data centers, high-performance computing and sensing^1^. Devices, that require the evanescent field of the guided mode to strongly interact with a cladding material, benefit from TM-polarization to maximize performance. Compared to the TE-mode, the evanescent field of the TM-guided mode outside the waveguide is enhanced. This can be exploited to increase the sensitivity of integrated evanescent field sensors^2–4^ and for nonlinear applications employing nonlinear waveguide claddings. The latter is a hybrid integration approach enabling on-chip nonlinear optics using high-intensity confined light and artificial materials with engineered optical nonlinearity^5^. TM-modes in this approach have been exploited for ultra-fast, integrated, electro-optic modulators^6,7^, and integrated non-reciprocal optical isolators^8,9^. Recently, a TM-mode-operated silicon-organic hybrid device has been proposed for ultra-efficient on chip THz generation^10^.

Coupling light from an optical fiber into submicron-sized silicon wire waveguides generally is a challenging task. A prevalent approach is to use grating couplers, since they provide the highest coupling efficiencies^11–17^. While grating couplers operating with TE-polarized light are well established, only a few examples for TM polarization have been presented in the literature^11,18–20^. Besides a high efficiency, grating couplers should ideally exhibit a compact geometrical footprint and a low-loss waveguide taper. Focusing grating lines with tailored curvature have been implemented in the grating coupler design to reduce the geometrical footprint of the coupler by tapering the grating itself^19,21^.

A challenge in designing any grating coupler lies in reducing back reflections between the grating and the connected waveguide. These reflections arise from the mismatch between the modes guided in the waveguide and the Bloch modes supported by the grating coupler region and may significantly contribute to the overall loss of the coupler. In addition, reflections cause unwanted Fabry-Perot resonances in the waveguides that may obscure the spectral characteristic of the transmission signal used to analyze the connected waveguide system. In regular fully-etched gratings, reflection coefficients are on the order of 30–40% (reflectivity: 9–17%) which results in Fabry-Perot ripples with an extinction ratio of 3 dB^19^. Back reflected fields may also feedback with engineered fields in the connected waveguide device, thus impeding optimal device functionality and operation, particularly in active nonlinear devices.

Special fabrication techniques have been proposed to mitigate back reflections. One way to overcome these reflections is to use shallow etched grating coupler designs^11–13^. In this approach, the mode mismatch is reduced, due to the fact that the grating has a weaker modulation and the Bloch modes have a higher group velocity and less back-propagating fields. Reflections in these designs are on the order of 0.6%^19^. For the case of resonant vertical coupling, a standing wave in the grating is excited and reflections can be higher even in shallow etched designs. A chirped grating section has been employed to reduce the reflections in this case^22^. In general, shallow-etched designs have the inherent disadvantage that the grating coupler and the connected waveguide structure cannot be defined in the same etching process. The shallow-etching step at least doubles the fabrication time compared to fully-etched designs and may result in additional time consuming optimization steps^19^. For fast device prototyping, a single etching step therefore represents the ideal fabrication process.

Alternatively to shallow etched designs, subwavelength high-index inclusions in between the grating lines were proposed^23^ and realized^18,19,21,24,25^ that also reduced a reflection. The designs are compatible with fully etched approaches^18,19,21,24,25^. The inclusions can be used to adjust the scattering strength of the grating for an optimal overlap with the fiber mode^21^. Additionally, they decrease a reflection by reducing the refractive index contrast in the grating and thus back propagating field components of the Bloch modes, similar to shallow etched designs. Reflections at the input of the grating in these designs are comparable to that in shallow-etched designs^19,23^. Subwavelength fully etched grating couplers for TM-modes with an insertion loss of 3.7 dB and a 3 dB bandwidth of 81.5 nm were recently realized^19^. However, feature sizes below 100 nm are incompatible with lithographic deep-UV fabrication processes. Even for electron-beam lithography, the small feature sizes are challenging to realize, particularly in optimized grating coupler designs employing apodized grating lines that require a high accuracy and only allow for small fabrication tolerances^21,25^.

Here, we investigate a photonic crystal anti-reflection approach for the design of fully-etched TM grating couplers with high coupling efficiency and low back reflections between the grating and the waveguide. We show that back reflections in any fully etched coupler are reduced to a minimum by a simple measure: making an exception for the width of the first slot in the grating.

Modified slots have been used by us^26^ and other groups^27^ before, however an analytic model for the approach has not been presented. Here we build upon this work and provide the generalized theory that enables fast and educated anti-reflection design of grating couplers. The approach is fundamentally different to existing design approaches with low back reflections, such as shallow-etched designs or designs employing subwavelength grating structures, in the sense that the anti-reflection boundary approach does not modify the Bloch mode of the grating to reduce the reflection. Instead, it rests on modifying the waveguide-grating interface only. We show that the approach is valid for arbitrary refractive index contrast in the grating. Therefore, it can be transferred to any grating coupler with fully etched slots as well as to other dielectric waveguide platforms such as silicon nitride^28^, titanium dioxide^6^, amorphous silicon on insulator^7^ and polymer sol-gel hybrids^29^. Our model shows that it also applies equally to the case of TE and TM modes. Using the approach, we optimize a TM-grating coupler for a wavelength of 1.55 µm and SOI wafers with a standard silicon thickness of 220 nm and a low refractive index cladding material (*n*_cladding_ = 1.7), as is required for nonlinear silicon-organic hybrid applications^5^. By means of focusing grating lines^19^, the coupler itself is tapered down to a width of 430 nm at a compact geometrical footprint. The strongly-tapered design with an opening angle of *α* = 26° avoids losses apparent in long adiabatic tapers of TM-waveguides^30^. The coupling loss and anti-reflection properties of the realized coupler are comparable to more sophisticated shallow-etched designs requiring multiple etching steps or designs employing subwavelength grating lines with small feature sizes. (Note that this comparison does not take into account specialized layer structures such as bottom reflector and overlay geometries^15,31–33^.) The coupler has fully etched slots and feature sizes above 200 nm and thus does not suffer from practical fabrication impairments enabling fast and practical integrated device prototyping.

## Backscattering design for suppression of back reflections

**Figure 1:**
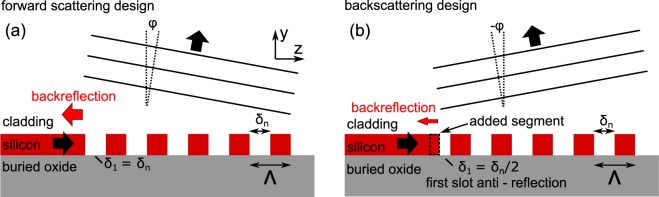
(**a**) Forward scattering design of a grating coupler. The grating is excited with a frequency above the photonic band gap. Thus, the coupler scatters into a positive scattering angle *φ*. The adjustment of the first slot in the grating cannot prevent a reflection at the waveguide-to-grating-boundary. (**b**) Backscattering design investigated in this work. Light from the waveguide is coupled at a frequency below the photonic band gap of the grating and is therefore scattered under a negative angle *φ*. The first slot of the grating is modified to avoid a reflection in this case. Half of the nominal slot width *δ* is filled with silicon.

We first introduce the “backscattering” approach for a grating coupler that ideally leads to a fully-etched designs with low back reflections. Figure 1(a,b) show 2D cross sections of grating couplers in a forward scattering design and in the proposed backscattering design, respectively. In the forward scattering design, a silicon waveguide couples radiation into the grating region at a frequency above the photonic band gap (PBG). Thus, light is scattered into a positive scattering angle *φ* which represents the conventional design approach for grating couplers^11–17^. No design exception is made for the width of the first slot in the grating *δ*_1_. The red arrow indicates back-reflections between the grating coupler and the waveguide. Such back reflections are caused by the mode mismatch of the waveguide mode and the modes supported in the grating coupler region. They can represent a substantial loss mechanism^19^ and cause Fabry-Perot ripples in the transmission signal. Figure 1(b) shows the proposed backscattering scheme for the design of a grating coupler. The light is coupled into the grating with a frequency below the PBG. Consequently, radiation from the waveguide is scattered in the grating under a negative angle *φ* which we refer to as “backscattering design”. We show that, in the backscattering design, reflections between the waveguide and the grating can be eliminated when the waveguide is positioned closer to the grating such that half of the first slot of the grating in filled with silicon as shown in Fig. 1(b). In this case *δ*_1_/*δ*_n_ = 0.5, where *δ*_1_ denotes the width of the first slot and *δ*_n_ the width of all other slots. The first slot adjustment suppresses reflections only in the backscattering design (i.e. negative scattering angle *φ*) as we detail in the Supplementary Information. Subwavelength inclusions or shallow etched grating lines are not required to suppress reflections. The negative scattering angle does not impose a problem for practical coupling setups as the fiber can also be tilted as is required in many setups. In the Supplementary Information we model the grating as a one-dimensional photonic crystal and present the band diagram. We discuss why the excitation below and above PBG leads to different signs of the scattering angle and derive the anti-reflection condition (*δ*_1_/*δ*_n_ = 0.5) according to which the waveguide is positioned with respect to the grating. The approach is valid for TM- and TE-polarization. It is also valid for arbitrary refractive-index contrast in the grating making it transferrable to other waveguide platforms. It is compatible with focusing grating lines as we also demonstrate in this paper. Irrespective of polarization and index contrast, the condition to suppress reflections in the backscattering design is always given for *δ*_1_/*δ*_n_ = 0.5 as illustrated in Fig. 1(b)).

**Figure 2:**
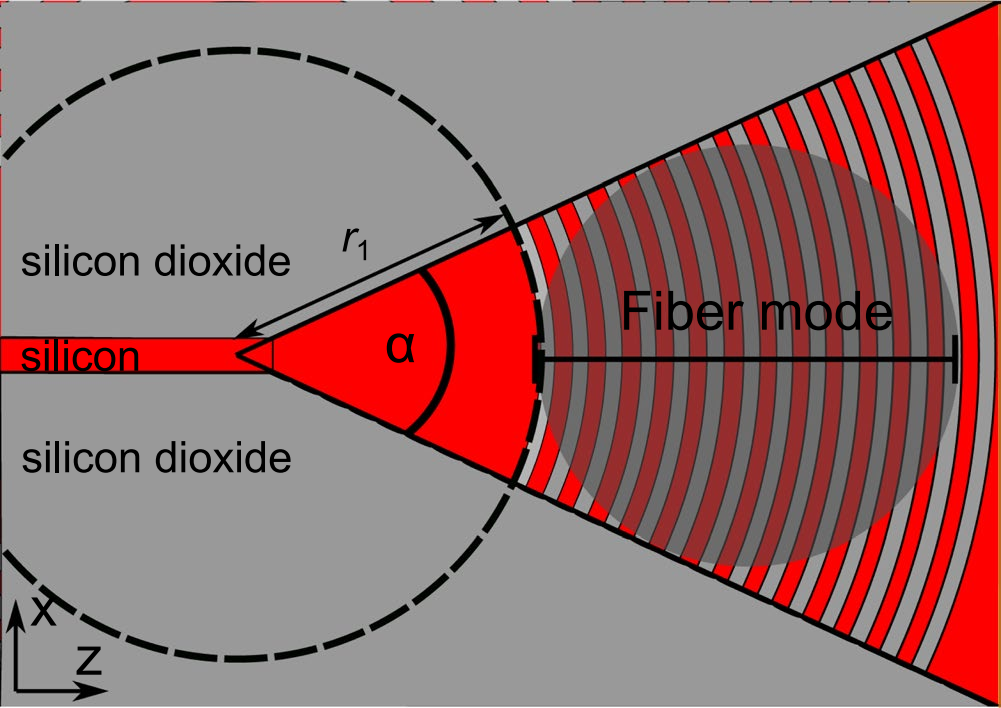
Schematic top view of the tapered grating coupler. The grating lines have a circular curvature as is indicated by the dashed circle. The fiber mode with 10 µm beam diameter fits inside the wedge as indicated by the gray transparent circle. The opening angle of the wedge is *α* = 26°.

## Design of a focusing TM grating coupler with anti-reflection boundary

We now use the backscattering design approach to realize a fully-etched grating coupler for TM-modes with low back reflections and focusing properties on the silicon photonics platform. The grating coupler is optimized for a silicon-on-insulator chip with a polymer cladding with a refractive index of 1.72, corresponding to a standard electro optic polymer employed in nonlinear optics applications^5,34^. We note however that the approach can be applied to any dielectric waveguide platform and arbitrary refractive index contrast in the grating. The first slot of the grating coupler *δ*_1_ is reduced to *δ*_n_/2 according to the anti-reflection design rule presented in the Supplementary Information. To optimize the remaining grating parameters, we conduct numerical simulations. We launch a waveguide mode at a frequency of 193 THz (/1.55 µm) into the waveguide and analyze the field pattern emitted into free space. The parameters of the grating coupler are optimized as follows^26^: First, we control the scattering angle of the coupler by adjusting the grating period *Λ*. Here, *Λ* is adjusted to yield a scattering angle into air *φ* = −10° as shown in Fig. 1(b). This corresponds to an emission angle of −6° in the polymer cladding according to Snell’s law. An angle of 10° in air is a convenient angle in most grating coupler setups, as it allows one to position the fiber closely to the grating. Second, after adjusting *Λ*, the slot width *δ*_n_ (determining the filling fraction of the grating $(\Lambda -{\delta }_{{\rm{n}}})/\Lambda$) is optimized. *δ*_n_ governs the spatial intensity profile of the light scattered from the coupler. We optimize *δ*_n_ such that the scattering intensity decreases to a value of 1/e within a distance of 8 µm. This value is the optimum for maximizing the coupling efficiency in a non-chirped design as it provides the maximal overlap between the scattered fields and the fiber mode profile of a standard telecom single mode fiber^26^.

The optimized parameters are *Λ* = 775 nm *δ*_n_ = 391 nm, *δ*_1_ = *δ*_n_/2 = 195.5 nm. Note that in our design, *δ*_n_ is not optimized to reduce a reflection since the adjustment of the first slot *δ*_1_ = *δ*_n_/2 reduces a reflection for any *δ*_n_ (i.e. filling fraction).

**Figure 3:**
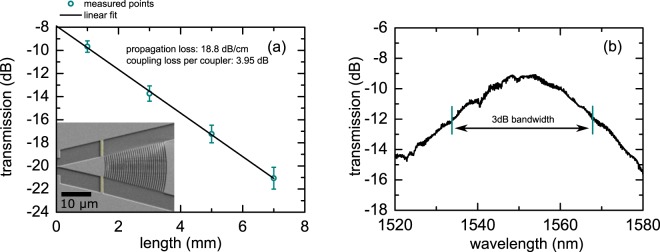
(**a**) Measured transmission loss for a waveguide with connected grating couplers on both ends as a function of waveguide length. The waveguide propagation loss is 18.8 dB/cm and the coupling loss per coupler is 3.95 dB. The inset shows a scanning electron micrograph of a fabricated coupler. The bridges (yellow false color) are electrical contacts that can be used to electrically contact the waveguide. Our simulations show that the effect of these contacts on the optical properties of the coupler is completely negligible. (**b**) Transmission spectrum for a waveguide with two grating couplers for 1 mm connecting waveguide.

For the taper, we choose a wedge design in which the grating itself focuses the light into the silicon wire waveguide with a width *d*_WG_ = 430 nm, as shown in Fig. 2. The wedge opening angle *α* is 26°. In the design, the grating pattern follows circular lines as shown by the dashed circle in Fig. 2. The center of the circles is positioned at the tip of the wedge which intersects the waveguide. In our taper design, we neglect curvature corrections to the circular grating trajectories that have been used in shallow etched designs with low refractive index contrast in the grating to optimize the focusing^21^. The grating starts in the wedge after a distance of *r*_1_ = 17.22 μm from the waveguide input. This configuration allows a full overlap between the grating and the fiber mode while maintaining a compact geometrical footprint as shown in Fig. 2. The inset in Fig. 3 (a) shows an SEM image of the realized coupler. The bridges (yellow color) are electronic contacts for the waveguide that do not affect the optical properties of the coupler as our simulations confirm.

## Results and Discussion

We experimentally determine the coupling loss of the fabricated grating couplers by a standard cut-back technique which enables one to distinguish between coupling loss of the grating coupler and propagation loss in the waveguide. In this approach, the transmission through waveguides of different length is measured where grating couplers are used on both ends of the waveguide for in and out-coupling of the light from a fiber-based setup. Figure 3(a) shows the transmission for waveguides of different length at a wavelength of 1.55 µm. The dots indicate measurement results from different samples, where the error bars correspond to the 1 sigma interval of the statistical distribution obtained from repetitive measurements of the same structures. As can be seen, the transmission on the log scale decreases linearly with the waveguide length as is expected for a homogenous waveguide. From the slope of the linear fit, we determine a propagation loss of (18.8 ± 0.7) dB/cm. A high propagation loss at wavelength of 1.5 µm for narrow TM wire waveguides is expected as a consequence of increased surface roughness scattering at the waveguide side walls^35^. From the intersection of the linear fit with the y-axis, we determine a coupling loss of (3.95 ± 0.15) dB per coupler. We experimentally determine a 3 dB bandwidth of 43 nm as shown in Fig. 3(b).

### Numerical analysis of the taper

**Figure 4:**
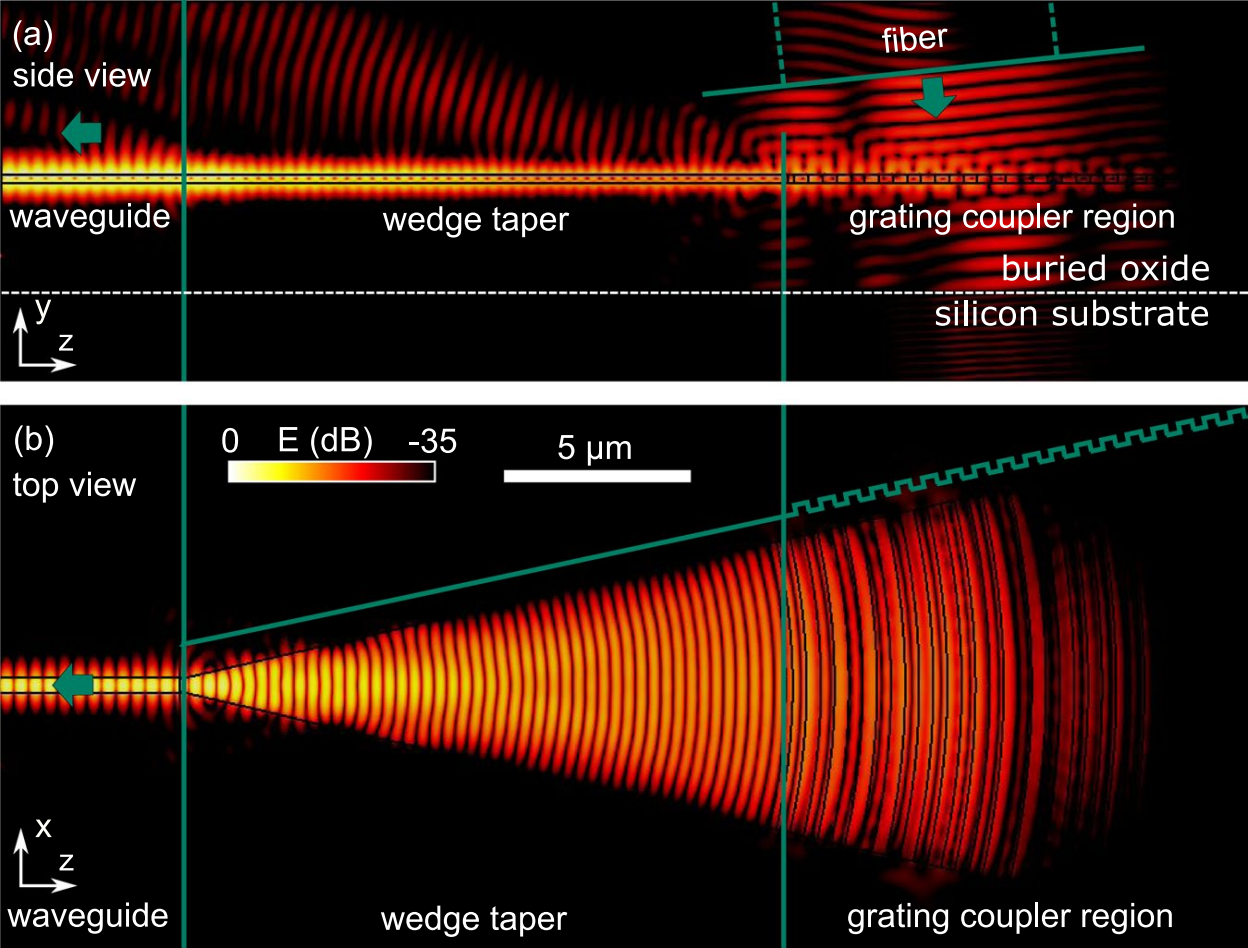
Numerical simulation of transmission from a Gaussian beam into the silicon wire waveguide in the proposed design. (**a**) Spatial electric field distribution from the side view. (**b**) Spatial electric field distribution from the top view.

We now further investigate the designed coupler in numerical simulations to analyze the focusing ability of the tapered design, the mode cross and the anti- reflection properties. Our simulations well support our experimental results and confirm the focusing ability of the design: We conduct three dimensional simulations of the full realized structure, including the waveguide and the tapered grating coupler and substrate material as shown in Fig. 4(a,b). The width of the first slot of the grating is adjusted according to the anti-reflection condition *δ*_1_/*δ*_n_ = 0.5. To analyze the focusing properties of the design, we excite the grating coupler with a TM-polarized Gaussian beam that represents the fields emitted from the fiber placed in close proximity to the grating coupler. As shown in Fig. 4(a), the Gaussian beam has a diameter of 10 µm which is comparable to the mode of a single mode fiber and is excited directly in the polymer cladding with an incident angle *φ* = −6°, corresponding to the coupling angle −10° in air, according to Snell’s law, for which the grating is optimized. The electromagnetic field distribution shown in Fig. 4(b) reveals that the excited fields are indeed well focused into the silicon waveguide by the curved grating lines. In the side view (Fig. 4(a)) we observe that fields are slightly scattered at the transition between the grating and the wedge and at the transition between the taper and the waveguide. Note that the intensity of these fields is low on the logarithmic color scale.

**Figure 5:**
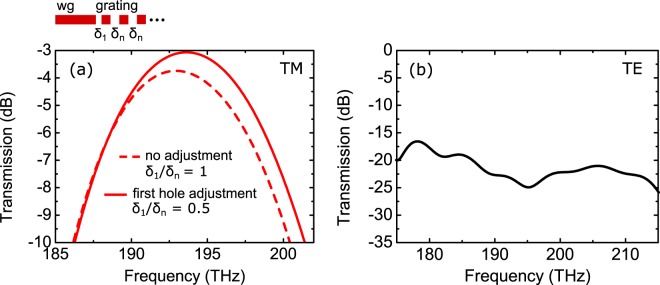
Simulated transmission from a Gaussian beam into the waveguide. The Gaussian beam is incident on the grating coupler as shown in Fig. 4(a). (**a**) Transmission from a TM-polarized Gaussian beam into the first order TM-mode of the silicon wire waveguide. The dashed line shows the case where no measure is taken to reduce a reflection (i.e. the width of the first slot is unaltered, *δ*_1_ = *δ*_n_. (**b**) Transmission of a TE polarized Gaussian beam into the first order TE-mode of the waveguide.

Figure 5(a) shows the simulated transmission from the Gaussian beam into the waveguide as a function of the excitation frequency. The coupling loss from the TM-polarized Gaussian beam into the TM-mode of the waveguide is 3.09 dB at 193 THz (1.55 µm). This well supports our experimental result (3.95 ± 0.15) dB. As a reference we also plot the simulated case where no measure is taken to reduce a reflection (i.e. the width of the first slot is unaltered, *δ*_1_ = *δ*_n_). In this case the coupling loss is increased from 3.09 dB to 3.8 dB. For the TE-polarized Gaussian beam coupled into the TE-mode, the loss is between 17 and 25 dB in the investigated frequency range is shown in Fig. 5(b). The TE-polarization has its peak transmission at a frequency of 165 THz (1.82 µm) with a coupling loss of 5.9 dB for the investigated coupling angle of *φ* = −10° (data not shown in Fig. 5(b)). Our simulations also reveal that TE- and TM-modes do not cross talk in the tapered grating: for excitation with a TM-polarized Gaussian beam, the excitation of the TE-mode in the waveguide is suppressed to below −100 dB. The same result is obtained for transmission from a TE-polarized Gaussian beam into the TM-mode of the waveguide. The low cross talk is the consequence of the strongly tapered design with a wedge opening angle of *α* = 26°.

### Numerical analysis of anti-reflection design

**Figure 6:**
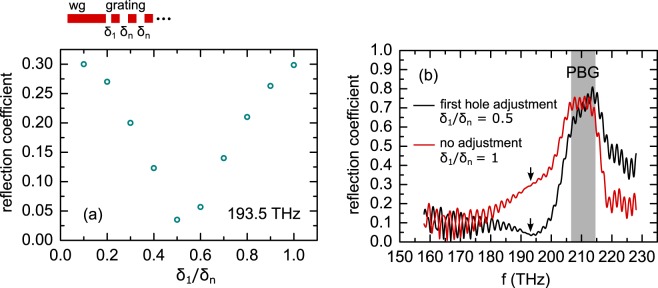
(**a**) Reflection coefficient in dependence on the width of the first slot in the grating *δ*_1_/*δ*_n_ for the operation frequency 193.5 THz. (**b**) Reflection coefficient for the proposed grating coupler with and without photonic crystal anti-reflection design. Arrows indicate the operation frequency at 193.5 THz.

In this section we investigate the anti-reflection properties of the coupler. For this purpose we now excite the TM-mode in the waveguide and probe the signal back reflected from the tapered grating coupler into the waveguide. Again we use three-dimensional simulations of the full geometry as shown in Fig. 4. We first confirm that the anti-reflection design yields the minimum back-reflection coefficient, as reported in Fig. 6(a). The graph shows the dependence of the reflection coefficient on the width of the first slot *δ*_1_/*δ*_n_ at the optimized operation frequency of 193.5 THz (/1.55 µm). We note the presence of a minimum if the first slot has half the width of all other slots (*δ*_1_/*δ*_n_ = 0.5), providing a reflection coefficient below 4% (reflectivity 0.16%). This result is consistent with the theoretical analysis presented in the Supplementary Information, where we model the grating as a one-dimensional photonic crystal stack consisting of alternating air and dielectric layers. We show that in this model, the minimum reflection is indeed expected for *δ*_1_/*δ*_n_ = 0.5 for an arbitrary refractive index contrast in the grating.

Furthermore, when employing *δ*_1_/*δ*_n_ = 0.5 we observe a suppression of the reflection down to values of 4% (reflectivity 0.16%) over a broad frequency range below the PBG as shown in Fig. 6(b). For the frequencies above the PBG, the proposed design increases reflection, which is however irrelevant since the devices are not operated in this frequency range. For comparison, we also analyze the design without an anti-reflection section (i.e. the width of the first slot is unaltered, δ_1_/δ_n_ =1). In this case, back reflections at 193 THz (/1.55 µm) are indeed substantial (30%) and represent a significant loss mechanism in the coupler and cause unwanted Fabry-Perot resonances in the waveguide.

## Conclusion

In conclusion, we propose to use an anti-reflection boundary approach for our novel backscattering design of a focusing grating coupler with fully etched slots for TM-polarization. In our design approach, light is excited at a frequency below the photonic band gap of the grating and thus is scattered under a negative angle. Back reflections between the waveguide and the grating are suppressed when half of the first slot in the grating is filled, i.e. *δ*_1_/*δ*_n_ = 0.5. Using the approach, we designed and realized a tapered TM grating coupler on a silicon-on-insulator platform with polymer-cladding. We experimentally determine a coupling loss of 3.95 dB at the telecom frequency of 193 THz (1.55 µm vacuum wavelength) and a 3 dB bandwidth of 43 nm. Three-dimensional simulations reveal that back reflections in the tapered design are indeed suppressed to 4% (reflectivity 0.16%). This is comparable to more sophisticated shallow etched and subwavelength grating coupler designs. The simulations also confirm the focusing properties of the circularly shaped grating lines and reveal that mode cross talk between the TM- and TE-mode in the taper is effectively suppressed. The design enables fast device prototyping since the slots are fully etched and the structure sizes are above 200 nm. We expect to improve these designs further by implementing a chirped (i.e. apodized) grating design^26^ and bottom reflectors^31^. The unique photonic crystal anti-reflection approach for design of a grating coupler only works in the backscattering design (i.e. employing a negative scattering angle *φ*). It is valid for TM- and TE-polarization and can be transferred to grating couplers on other waveguide platforms and other grating coupler materials. Irrespective of polarization and refractive-index contrast in the grating, the condition to suppress reflections is always given when half of the first slot in the grating is filled, i.e. *δ*_1_/*δ*_n_ = 0.5.

## Materials and Methods

### Sample preparation

Electron beam lithography and a single-step reactive ion etch are used to fabricate the designed grating couplers on SOI wafers with a silicon layer of 220 nm thickness and a box thickness of 3 µm. Subsequently, samples are coated with a polymer cladding^34^, based on a chromophore doped amorphous polycarbonate, with a refractive index of 1.7. Such claddings can be used for nonlinear optical applications where the high field overlap of the TM-mode with the cladding shall be exploited^6–10^.

### Optical measurements

We experimentally determine the coupling loss of the fabricated grating couplers by a standard cut-back technique which enables one to distinguish between coupling loss of the grating coupler and propagation loss in the waveguide. In this approach, the transmission through waveguides of different length is measured where grating couplers are used on both ends of the waveguide for in and out-coupling of the light from a fiber-based setup. We use a setup with adjustable fiber angle and a tunable polarized continuous-wave laser source with an operation wavelength in the 1.55 µm C-band. The polarization of the light coupled to the waveguide is controlled using fiber-based polarizers. Coupling is optimized by maximizing the transmission signal.

### Numerical simulations

We use the commercial software CST microwave studio (www.cst.com) which employs the finite-integration-technique. Numerical simulations of electromagnetic field distributions, transmission, and reflection are carried out in the time domain.

## Electronic supplementary material

Supplementary Information:

## Data Availability

The authors declare that the data supporting the findings of this study are available within the article and its Supplementary Information file. The raw measurement data are available from the corresponding author on reasonable request.

## References

[1] Thomson D (2016). Roadmap on silicon photonics. J. Opt..

[2] Densmore A (2006). A Silicon-on-Insulator Photonic Wire Based Evanescent Field Sensor. IEEE Photon. Technol. Lett..

[3] Xu D-X (2008). Folded cavity SOI microring sensors for high sensitivity and real time measurement of biomolecular binding. Opt. Express.

[4] Fohrmann LS (2017). Integrating cell on chip—Novel waveguide platform employing ultra-long optical paths. APL Photonics.

[5] Koos C (2016). Silicon-Organic Hybrid (SOH) and Plasmonic-Organic Hybrid (POH) Integration. J. Lightwave Technol..

[6] Qiu F (2016). An electro-optic polymer-cladded TiO2 waveguide modulator. Appl. Phys. Lett..

[7] Sato H (2017). Low driving voltage Mach-Zehnder interference modulator constructed from an electro-optic polymer on ultra-thin silicon with a broadband operation. Opt. Express.

[8] Stadler BJH, Mizumoto T (2014). Integrated Magneto-Optical Materials and Isolators: A Review. IEEE Photon. J..

[9] Qi W, Jin Y, Yu H, Jiang X (2015). A magneto-optical isolator based on series-coupled race-track resonators. ‎Jpn. J. Appl. Phys.

[10] Schulz K. M., Rusche A. G. C., Petrov A. Yu., Eich M. (2018). Integrated Nonlinear Waveguide Optics for High-Efficiency and Wideband-Tunable Generation of THz Radiation. ACS Photonics.

[11] Zaoui WS (2014). Bridging the gap between optical fibers and silicon photonic integrated circuits. Opt. Express.

[12] Passoni M, Gerace D, Carroll L, Andreani LC (2017). Grating couplers in silicon-on-insulator: The role of photonic guided resonances on lineshape and bandwidth. Appl. Phys. Lett..

[13] Taillaert D (2006). Grating Couplers for Coupling between Optical Fibers and Nanophotonic Waveguides. Jpn. J. Appl. Phys.

[14] Cheng Z, Li Z, Xu K, Tsang HK (2014). Increase of the grating coupler bandwidth with a graphene overlay. Appl. Phys. Lett..

[15] Xiao Z, Luan F, Liow T-Y, Zhang J, Shum P (2012). Design for broadband high-efficiency grating couplers. Opt. Lett..

[16] Cheben P (2006). A broad-band waveguide grating coupler with a subwavelength grating mirror. IEEE Photon. Technol. Lett..

[17] Mekis A (2011). A grating-coupler-enabled CMOS photonics platform. IEEE J. Sel. Topics Quantum Electron..

[18] Halir R (2010). Continuously apodized fiber-to-chip surface grating coupler with refractive index engineered subwavelength structure. Opt. Lett..

[19] Wang Y (2014). Focusing sub-wavelength grating couplers with low back reflections for rapid prototyping of silicon photonic circuits. Opt. Express.

[20] Chen X, Tsang HK (2011). Polarization-independent grating couplers for silicon-on-insulator nanophotonic waveguides. Opt. Lett..

[21] Cheng Z, Chen X, Wong CY, Xu K, Tsang HK (2012). Apodized focusing subwavelength grating couplers for suspended membrane waveguides. Appl. Phys. Lett..

[22] Chen X, Li C, Tsang HK (2008). Fabrication-tolerant waveguide chirped grating coupler for coupling to a perfectly vertical optical fiber. IEEE Photon. Technol. Lett..

[23] Halir R (2009). Waveguide grating coupler with subwavelength microstructures. Opt. Lett..

[24] Liu L, Pu M, Yvind K, Hvam JM (2010). High-efficiency, large-bandwidth silicon-on-insulator grating coupler based on a fully-etched photonic crystal structure. Appl. Phys. Lett..

[25] Xu, X. *et al*. (eds). *CMOS compatible subwavelength grating couplers for silicon integrated photonics*. IEEE Photonics Conference 2012 (2012).

[26] Schmid B, Petrov A, Eich M (2009). Optimized grating coupler with fully etched slots. Opt. Express.

[27] Roelkens G, van Thourhout D, Baets R (2007). High efficiency grating coupler between silicon-on-insulator waveguides and perfectly vertical optical fibers. Opt. Lett..

[28] Gondarenko A, Levy JS, Lipson M (2009). High confinement micron-scale silicon nitride high Q ring resonator. Opt. Express.

[29] Enami Y (2007). Hybrid cross-linkable polymer/sol-gel waveguide modulators with 0.65V half wave voltage at 1550nm. Appl. Phys. Lett..

[30] Vermeulen, D. *et al*. Efficient tapering to the fundamental Quasi-TM mode in asymmetrical waveguides, *ECIO* (2010).

[31] Taillaert D, Bienstman P, Baets R (2004). Compact efficient broadband grating coupler for silicon-on-insulator waveguides. Opt. Lett..

[32] Roelkens G (2008). High efficiency diffractive grating couplers for interfacing a single mode optical fiber with a nanophotonic silicon-on-insulator waveguide circuit. Appl. Phys. Lett..

[33] Vermeulen D (2010). High-efficiency fiber-to-chip grating couplers realized using an advanced CMOS-compatible Silicon-On-Insulator platform. Opt. Express.

[34] Schulz KM (2015). Mechanism that governs the electro-optic response of second-order nonlinear polymers on silicon substrates. Opt. Mater. Express.

[35] Vlasov YA, McNab SJ (2004). Losses in single-mode silicon-on-insulator strip waveguides and bends. Opt. Express.

