# Supplementary material for: Backscattering design for a focusing grating coupler with fully etched slots for transverse magnetic modes
Source: Sci Rep. 2018 Dec 10;8:17746. doi: 10.1038/s41598-018-36082-z (PMC6288090; doi:10.1038/s41598-018-36082-z)
Supplement: Supplementary file 1 — Supplementary Information: [file 41598_2018_36082_MOESM1_ESM.pdf]

Supplementary Information

Backscattering design for a focusing grating coupler with fully etched slots for transverse magnetic modes

Jahn Hoffmann[1], K. Marvin Schulz[1*], Giampaolo Pitruzzello[2], Lena Simone Fohrmann[1], Alexander Yu. Petrov[1,3] and Manfred Eich[1,4]

[1]Institute of Optical and Electronic Materials, Hamburg University of Technology, Hamburg 21073, Germany
[2]Department of Physics, University of York, York, YO10 5DD, UK
[3]ITMO University, 49 Kronverkskii Ave., 197101, St. Petersburg, Russia
[4]Institute of Materials Research, Helmholtz-Zentrum Geesthacht, Max-Planck-Strasse 1, Geesthacht, D-21502,Germany

[*] To whom correspondence should be addressed: marvin.schulz@tuhh.de

One dimensional photonic crystal with anti-reflection boundary. In the following, we show that a 1D photonic crystal anti-reflection boundary can be designed to suppress back reflections when coupling from homogenous space into a photonic crystal. We show that the anti-reflection condition is met when half of the first slot in the grating is filled (i.e. $\delta_1/\delta_n = 0.5$). A necessary condition to suppress reflections is that light is coupled into the dielectric mode of the photonic crystal slab, that is, the mode below the band gap with energy mainly concentrating in the dielectric material. This mode scatters light with a negative angle φ into free space as shown in Fig. 1(b) of the main text.

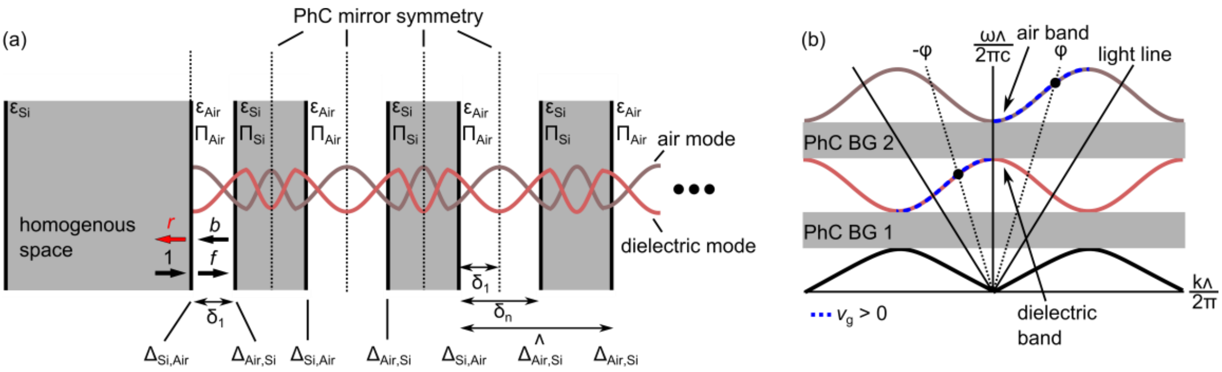

Fig. S1. (a) A wave with a normalized amplitude of 1 impinges from homogenous space onto a photonic crystal slab where it couples into supported Bloch modes. A part of this wave is back reflected with an amplitude r. Brown solid line: schematic electric field energy density distribution of the "air" mode at a frequency above the second bandgap of the photonic crystal. Red solid line: electric field energy density distribution of the "dielectric" mode at a frequency below the bandgap. (b) Schematic photonic crystal band diagram. Modes above the light line scatter light into the air under a certain scattering angle φ determined by the photonic crystal dispersion. Only modes with group velocity $v_g > 0$ can be excited from the direction of the waveguide (dashed blue line).

Fig. S1(a) shows the considered geometry. A wave incident from homogenous space couples into Bloch modes in a photonic crystal. The incident wave has a normalized amplitude of 1. The Bloch mode in every layer of the stack can be described by a superposition of forward

and backward running plane waves denoted by their amplitudes as f and b. Back reflections into the homogenous medium, that shall be mitigated by our approach, are denoted as r. When coupling from the homogenous medium into the photonic crystal, certain modes can be excited that obey the photonic crystal dispersion relation schematically shown in Fig. S1(b). Generally, bands located above the light line can scatter light into free space. The relevant bands are the air and dielectric band at the second photonic crystal bandgap (PhC BG 2) as indicated in Fig. S1(b). Modes located at the first band gap do not have to be considered since they lie below the light line and do not scatter light to free space. In Fig. S1(a) we show schematically the electric field energy density distribution of the modes close to the second bandgap. We note that compared to the air mode, the dielectric mode concentrates a larger percentage of electric field energy in the silicon. We also remark that these modes accumulate a phase of 2π within the lattice constant Λ since they are located at the second bandgap.

We now show that reflections r can be mitigated when coupling to the dielectric band of the photonic crystal. Following a transfer matrix approach, a system of linear equations can be formulated to describe the coupling from homogenous space into a photonic crystal[1,2]

$$\begin{pmatrix} 1 \\ r \end{pmatrix} = \Delta_{\mathrm{Si,Air}} t_{\mathrm{B}} \begin{pmatrix} f \\ b \end{pmatrix} \qquad (1)$$

with $\Delta_{\mathrm{Si,Air}}$ being the transition matrix at the silicon/air boundary defined as $\Delta_{\mathrm{Si,Air}} = \frac{1}{t_{\mathrm{Si,Air}}} \begin{pmatrix} 1 & r_{\mathrm{Si,Air}} \\ r_{\mathrm{Si,Air}} & 1 \end{pmatrix}$. $t_{\mathrm{Si,Air}}$ and $r_{\mathrm{Si,Air}}$ are the Fresnel transmission and reflection coefficient for normal incidence given by

$$t_{\mathrm{Si,Air}} = \frac{2n_{\mathrm{Si}}}{n_{\mathrm{Si}} + n_{\mathrm{Air}}} \qquad (2)$$

and

$$r_{\mathrm{Si,Air}} = \frac{n_{\mathrm{Si}} - n_{\mathrm{Air}}}{n_{\mathrm{Si}} + n_{\mathrm{Air}}}, \qquad (3)$$

where $1{+}r_{\mathrm{Si,Air}} = t_{\mathrm{Si,Air}}$. Note that for normal incidence these coefficients are polarization independent. t_B is the transmission coefficient of the Bloch mode described by a vector (f, b). The Bloch mode is normalized by its power as $|f|^2 - |b|^2 = 1$.

Solving Eq. (1) for r yields

$$r = t_B \frac{1}{t_{\mathrm{Si,Air}}} \left(f r_{\mathrm{Si,Air}} + b \right) . \tag{4}$$

In the case of suppressed back reflections, $r = 0$, and it follows that $f r_{\mathrm{Si,Air}} + b = 0$ since $t_B \frac{1}{t_{\mathrm{Si,Air}}} \neq 0$. Thus, we can write the condition for $r = 0$ as

$$r_{\mathrm{Si,Air}} = -\frac{b}{f} \tag{5}$$

We note that $r_{\mathrm{Si,Air}}$ is real valued according to Eq. (3). Therefore b and f are either also real valued or must have the same phase. In the following we show that b and f are always real valued at locations in the photonic crystal slab that satisfy spatial mirror symmetry. Mirror symmetry is always provided in the middle of the dielectric and air layers of the photonic crystal slab as indicated in Fig. S1(a). This mirror symmetry is the physical origin for the final design rule $\delta_1/\delta_{\mathrm{n}} = 0.5$ for reducing back reflections.

The eigenvalue equation for the 1D photonic crystal slab can be written as [3]

$$e^{-ik_b\Lambda} \begin{pmatrix} f \\ b \end{pmatrix}_{z+\Lambda} = M^{\Lambda} \begin{pmatrix} f \\ b \end{pmatrix}_{z+\Lambda}, \tag{6}$$

where M^{Λ} is the product of transition Δ and propagation matrices Π given by $M^{\Lambda} = \Pi_1 \Delta_{\mathrm{Si,Air}} \Pi_2 \Delta_{\mathrm{Air,Si}} \Pi_3 \ldots$, where Π is given by $\Pi[\delta] = \begin{pmatrix} e^{ik\delta} & 0 \\ 0 & e^{-ik\delta} \end{pmatrix}$ with δ being the propagation distance. Λ is the lattice constant of the photonic crystal.

The complex conjugate of Eq. (6) is

$$e^{ik_b\Lambda} \begin{pmatrix} f^* \\ b^* \end{pmatrix}_{z+\Lambda} = M^{\Lambda *} \begin{pmatrix} f^* \\ b^* \end{pmatrix}_{z+\Lambda} \tag{7}$$

which can be rearranged to

$$M^{\Lambda*-1}\begin{pmatrix} f^* \\ b^* \end{pmatrix}_{z+\Lambda} = e^{-ik_b\Lambda}\begin{pmatrix} f^* \\ b^* \end{pmatrix}_{z+\Lambda}. \tag{8}$$

It can be shown that $M^{\Lambda*-1} = M^{\Lambda}$ if $\Pi_1 = \Pi_3$, which is fulfilled when the unit cell is considered between two mirror symmetry planes. Therefore Eq. (8) can be written as

$$M^{\Lambda}\begin{pmatrix} f^* \\ b^* \end{pmatrix}_{z+\Lambda} = e^{-ik_b\Lambda}\begin{pmatrix} f^* \\ b^* \end{pmatrix}_{z+\Lambda} \tag{9}$$

Comparison of Eq. (9) and Eq. (6) leads us to conclude that $\begin{pmatrix} f^* \\ b^* \end{pmatrix} = \begin{pmatrix} f \\ b \end{pmatrix}$ and hence f and b are real valued at locations with mirror symmetry.

Thus, we will now consider the case that $-b/f$ is real. As we have shown, this condition is fulfilled if the first air layer is terminated at half of the width of all other air layers (i.e. $\delta_1/\delta_n = 0.5$) since it satisfies mirror symmetry at this position. $r_{\mathrm{Si,Air}} > 0$ according to Eq. (3) since we couple from a high refractive index medium into a multilayer stack that starts with a low refractive index medium as shown in Fig. S1(a). The case $r_{\mathrm{Si,Air}} < 0$ does not have to be considered since it corresponds to the case where we couple from a low refractive index medium into the grating that starts with a high refractive index medium. This case is not given when coupling from the waveguide into the grating.

For $r_{\mathrm{Si,Air}} > 0$ it follows that $b/f < 0$ according to Eq. (5) meaning that b and f are out of phase in the air layer. This situation is only obtained when coupling to the dielectric mode of the photonic crystal which has out-of-phase components in air and consequently a low intensity in air. We remark that it cannot be fulfilled for the air mode since this mode has in-phase components in air and thus $b/f > 0$. As shown in the band diagram in Fig. S1 (b), coupling to the dielectric mode results in a negative scattering angle φ since we can only couple to modes with a positive group velocity $v_{\mathrm{g}} > 0$ when exciting from the direction of the waveguide (i.e. homogenous space).

In the last step it is important to match b/f and $r_{\mathrm{Si,Air}}$ in order to suppress r according to Eq. (5). This matching happens automatically close to the band edge. At the band edge $\frac{b}{f} = 1$ since modes are standing waves. However, b/f reduces from a value of 1 away from the band edge. Thus, there exists a frequency where the reflection r is exactly zero.

In conclusion, we emphasize that the anti-reflection boundary approach only works when coupling to the dielectric mode of the photonic crystal slab. Therefore, designs in the anti-reflection configuration necessarily result in a negative scattering angle φ. The approach equally works for TE- and TM-polarizations and any filling fraction of the grating $((\Lambda - \delta_n)/\Lambda)$ since no assumption on these parameters is made. The refractive index contrast in the photonic crystal slab is also arbitrary making the approach transferrable to other waveguide platforms. Irrespective of these parameters, the condition to suppress reflections is always given when half of the first slot in the grating is filled , i.e. $\delta_1/\delta_{\mathrm{n}} = 0.5$, representing a conceptually simple design rule.

References

1. Bendickson, J. M. Dowling, J. P. & Scalora, M. Analytic expressions for the electromagnetic mode density in finite, one-dimensional, photonic band-gap structures, *Phys. Rev. E* **53,** 4107–4121 (1996).
2. Petrov, A. *Slow light photonic crystal line-defect waveguides.* (PhD Thesis, Hamburg University of Technology, Hamburg, 2008).
3. Yariv, A. and Yeh, P. *Optical Waves in Crystals: Propagation and Control of Laser Radiation* (Wiley, New York, 1984).
